# The discourse of organizational resilience before and after the global pandemic

**DOI:** 10.12688/f1000research.133601.1

**Published:** 2023-09-11

**Authors:** Budi Harsanto, Egi Arvian Firmansyah

**Affiliations:** 1 Department of Management and Business, Universitas Padjadjaran, Bandung, West Java, 40132, Indonesia; 2 School of Business and Economics, Universiti Brunei Darussalam, Bandar Seri Begawan, Brunei-Muara District, Brunei

**Keywords:** discourse analysis, organizational resilience, pre and post-pandemic

## Abstract

**Background:** Over the past decade, there has been a surge in public and academic discussions about organizational resilience, particularly in the wake of the coronavirus disease (COVID-19) pandemic. It is essential to understand the changes in the discourse of organizational resilience before and after the pandemic. This study aims to understand how the concept of organizational resilience evolved before and after the pandemic.

**Methods:** This study is qualitative in nature, employing discourse analysis techniques on scholarly documents on organizational resilience. Our analysis considers the global context of organizational resilience discussions and highlights the most frequently discussed industries, such as tourism and hospitality, manufacturing, and healthcare. The documents were searched on Scopus academic databases with the key search term of organizational AND resilience.

**Results:** Our findings indicate that themes related to “response to external threats” and “supply chain vulnerabilities and disruptions” have gained significant attention post-pandemic. Meanwhile, discussions around “preparedness and organizational reliability” and “coping with occupational and job demands” have remained consistent before and after the pandemic.

**Conclusions:** This study contributes to the academic understanding and practical application of organizational resilience evolution by discourse before and after the pandemic. It highlights the significance of being prepared for external threats and managing supply chain disruptions while recognizing the importance of preparedness and organizational reliability.

## Introduction

The coronavirus disease (COVID-19) pandemic has profoundly impacted business activities and organizations worldwide ( Setiawan *et al.*, 2023). Many researchers in business and management fields have also documented various findings related to COVID-19, as presented in a study by Harsanto and Firmansyah (2023a). Furthermore, the concept of resilience, or the ability to endure adverse circumstances, has gained increased recognition and attention in the management field, particularly in the context of crisis management. The term resilience was first commonly used in reference to how well society can respond to and recover from natural disasters ( Bohensky & Leitch, 2014). Despite this increased attention, in the context of organizational resilience, the definition and understanding remain evolving and inconsistent, with different authors emphasizing different aspects such as capacity, capability, or outcomes ( Duchek, 2014; Hillmann & Guenther, 2021).

Organizational resilience is crucial in facing disruptions and challenges, as leaders must respond effectively to maintain and grow their businesses ( Denyer, 2017). The pandemic represents a severe test of a firm’s ability to adapt and become stronger in adverse situations. Firms need to quickly learn and adapt to seize opportunities, intending to bounce back effectively and avoid prolonged downturns, even if they experience short-term setbacks ( Teo *et al.*, 2017).

The concept of organizational resilience has gained more attention recently, particularly after the outbreak of the COVID-19 pandemic in 2020. However, there has been a lack of research comparing the development of the concept before and after the pandemic. The pandemic, an extraordinary event considered one of the worst global events since World War II, has significantly impacted organizations and their ability to adapt and bounce back in the face of adversity. Understanding how the discourse of organizational resilience has evolved before and after the pandemic would be valuable for academics and practitioners in the field. It would provide insights into how organizations have responded to the unprecedented challenges posed by the pandemic and how they have worked to enhance their resilience.

Therefore, this study aims to understand how the concept of organizational resilience evolved before and after the pandemic. The study seeks to answer the following research questions: (1) In which industries was the concept of organizational resilience used before and after the pandemic? (2) What themes in organizational resilience have received increased attention before and after the pandemic? (3) How has the discourse surrounding each theme changed before and after the pandemic?

The contribution of this study lies in its ability to provide an understanding of how the discourse of organizational resilience has evolved before and after the global pandemic. In addition, by examining the industries in which the concept was used and the themes that received increased attention, this study offers insights into how organizations have responded to the unprecedented challenges posed by the pandemic.

The implications of this study are significant for both academics and practitioners in the field of organizational resilience. For academics, the findings of this study can serve as a basis for further research and development of the concept. For practitioners, the insights from this study can be applied in practical settings to enhance their organization resilience and ability to respond to future disruptions. The results of this study can also inform public policy decisions aimed at improving the resilience of organizations in the wake of future pandemics or other disruptive events.

This paper is structured as follows: the next section describes the methodology used to search for datasets and the analytical techniques used. The study results are presented in the third section, followed by a discussion in the fourth section. The last section is implications and further directions.

## Methods

### Data collection

This study is qualitative in nature, employing discourse analysis techniques on scholarly documents of organizational resilience ( Leitch & Bohensky, 2014). Discourse analysis is one technique of literature review. Data in this study are academic documents published by publishing slots, such as journals, conference proceedings, and book chapters. The search for documents for this study’s dataset was conducted on July 5, 2022. The scholarly dataset in this study was obtained from the Scopus (RRID:SCR_022559) database. Scopus and Web of Science are two comprehensive academic databases that are currently market leaders. In this study, we utilized Scopus due to its comprehensive coverage, which is more extensive than other databases ( Harsanto, 2020; Harzing & Alakangas, 2016). Scopus is widely recognized as the world’s largest abstract citation database of scholarly literature ( Schotten *et al.*, 2017).

During our search, we used the search term “Organizational AND Resilience” so that both terms were inseparable. In the initial search using: “TITLE (organizational AND resilience),” 584 documents were retrieved. The TITLE was used to obtain articles relevant to the topic under investigation straightforwardly. Further refinement was performed using “TITLE (organizational AND resilience) AND (LIMIT-TO (SUBJAREA, “BUSI”) OR LIMIT-TO (SUBJAREA, “ECON”)),” which meant we focused only on the subject areas of Business and Economics, resulting in 273 documents ( Harsanto & Firmansyah, 2023b). Finally, after selecting only articles written in English, the search yielded 262 documents. Therefore, the inclusion criteria in this study were as follows:

• Articles mentioning the phrase ‘organizational resilience’ in the title.
• Articles included in business and economics areas.
• Articles in the English language.

**Figure 1.**
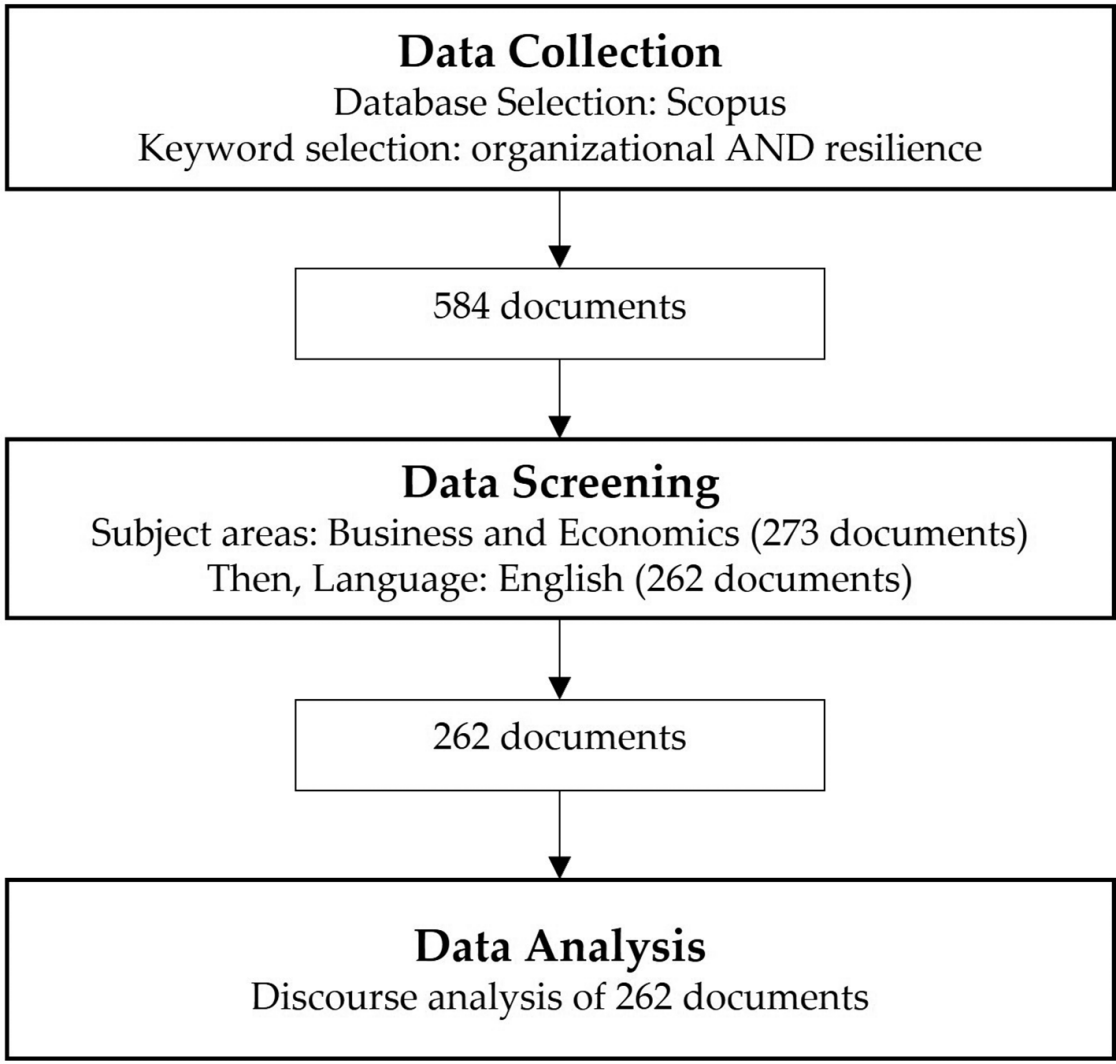
Methodological process.

Other articles not meeting those three criteria were excluded from this study. The overall methodological process is similar to previous studies ( Harsanto & Firmansyah, 2021; Jan *et al.*, 2022). The process is shown in Figure 1.

We manually read the article title and abstract to ensure that all documents were related to organizational resilience. Furthermore, limiting to business or economics subject areas was expected to provide better relevancy by resulting in only the documents discussing organizational resilience in business and economics settings. Omitting documents in other areas, such as engineering, medicine, and computer science, provided relevant documents in business and economics contexts. The obtained dataset (CSV format) was then converted and processed in an Excel spreadsheet for data cleaning and coding to produce research findings.

### Data analysis

The data of this study were analyzed in three different ways: descriptive statistical analysis, discourse analysis, and comparison analysis. First, descriptive statistics were used to understand two things: the industry context in which organizational resilience is applied ( Table 1) and document types ( Table 2). We used Microsoft Excel (RRID:SCR_016137) in Microsoft Office 365 software to produce these descriptive statistics, allowing us to tabulate each category. Abstract reading was performed to categorize the industry type, while Scopus data were used to categorize the document type of the selected documents.

**Table 1.** Organizational resilience terminology usage across industries.

Industry	No. of documents Before 2020	No. of documents 2020-2022	Total	% Total
Mixed	60	74	135	51.15%
Tourism & Hospitality	5	20	25	9.54%
Manufacturing	10	6	16	6.11%
Healthcare	4	10	14	5.34%
Others	3	10	13	4.96%
Service	6	4	10	3.82%
Education	3	6	9	3.44%
Public	5	2	7	2.67%
Food & beverages	3	3	6	2.29%
IT	2	3	5	1.91%
Retail	2	2	4	1.53%
Bank	1	2	3	1.15%
Non-profit	1	2	3	1.15%
Aviation	0	2	2	0.76%
Construction	2	0	2	0.76%
Electricity	1	1	2	0.76%
Textile	2	0	2	0.76%
Insurance	2	0	2	0.76%
Agricultural	1	0	1	0.38%
Furniture	1	0	1	0.38%
Pharmaceutical	1	0	1	0.38%

**Table 2.** Dataset by document type.

Document type	Frequency	Percentage
Article	198	75.57%
Book	7	2.67%
Book Chapter	21	8.02%
Conference Paper	16	6.11%
Editorial	4	1.53%
Letter	1	0.38%
Note	3	1.15%
Review	12	4.58%
Total	262	100.00%

In the second analysis, a discourse analysis, similar to Leitch and Bohensky, was used to examine the term “organizational resilience” in 262 documents. Discourse analysis is appropriate to select because it allows us to see the shift in organizational resilience studies before and after the pandemic. Specifically, discourse analysis provides information on how the term ‘organizational resilience’ is used in those two different points in time.

In the third analysis, which is the development of the second analysis, we mapped and compared the selected documents before and after the pandemic ( Leitch & Bohensky, 2014). We considered publications before 2020 to be before the pandemic, and 2020 onwards were after the pandemic. In particular, the analysis of the selected documents focuses on seven key themes of organizational resilience identified in Denyer’s report ( Denyer, 2017). We further produced a framework depicting the shift in the organizational resilience studies available in the literature between before and after the pandemic.

## Results

### Descriptive statistics for industry types

The term “organizational resilience” is used in different industries. As shown in Table 1 ( Harsanto & Firmansyah, 2023b), most of its use in previous studies was in mixed-industry analysis (51.15%), which involved more than one industry in one study. The three industries that individually receive the highest attention in studies on organizational resilience are tourism and hospitality (9.54%), manufacturing (6.115%), and healthcare (5.34%). The industry that experienced a rapid increase in academic attention when the pandemic hit was tourism and hospitality, which was indeed one of the industries that experienced the worst impact due to the imposition of restrictions on mobility and restrictions on activities involving many people.

### Descriptive statistics for document types

There have been diverse document types published in organizational resilience documents, as shown in Table 2. This study did not limit to only one or two types of documents because we expected to obtain heterogeneous information about organizational resilience from various publication types, thus enhancing the findings of this study. Furthermore, this study revealed that organizational resilience was primarily found in journal articles, with more than 75%. This percentage was higher than other types of research documents, such as conference papers (6.11%), books (2.67%), and book chapters (8.02%). It also indicated that organizational resilience had been documented in high-quality document types because journals require a peer-review process to filter the documents before publication.

### Descriptive and comparison results for the themes

Table 3 shows the percentage of publications on organizational resilience before and after the pandemic. The themes in the table were adopted from Denyer’s report on organizational resilience ( Denyer, 2017). From the analysis, it can be seen that both before and after the pandemic, the theme that received the highest attention was “preparedness and organizational reliability”, followed by “coping with occupational and job demands”. By contrast, the theme that received the least attention was “Ensuring IT/IS/cybersecurity”. It indicates that some studies are more general on organizational resilience or the impact on work in various areas of the organization instead of focusing on specific areas of IT with specific threats such as cyberattacks.

**Table 3.** Percentage of themes before and after the pandemic.

Themes	Before 2020		2020-2022		% Change	Sum	% of Sum
	No. of documents	%	No. of documents	%			
Response to external threats	5	4.35%	17	11.56%	7.22%	22	8.40%
Preparedness and organizational reliability	30	26.09%	39	26.53%	0.44%	69	26.34%
Coping with occupational and job demands	28	24.35%	37	25.17%	0.82%	65	24.81%
Renewal, strategic agility & crisis as opportunity	22	19.13%	20	13.61%	-5.52%	42	16.03%
Supply chain vulnerabilities and disruptions	6	5.22%	10	6.80%	1.59%	16	6.11%
Ensuring IT/IS/cyber security	5	4.35%	5	3.40%	-0.95%	10	3.82%
Defining and conceptualizing resilience	19	16.52%	19	12.93%	-3.60%	38	14.50%
Total	115	100.00%	147	100.00%		262	100.00%

Note: Themes adopted from Denyer (2017).

When comparing before and after the pandemic, the highest percentage increase was on the theme “Response to external threats,” followed by “Supply chain vulnerabilities and disruptions.” This is understandable because the pandemic, which was a great shock to organizations, made organizations worldwide struggle, and they were looking for the best response to these unexpected and highly impactful events. That makes publications regarding organizational responses to external threats has the highest percentage because these publications allow for observations of immediate impacts to be carried out directly. Among those severely shaken by the COVID-19 pandemic were supply chains due to lockdown policies, mobility restrictions, and other policies, especially at the beginning of the pandemic, which was full of uncertainty. The summary of organizational resilience discourse before and after the pandemic is shown in Table 4.

**Table 4.** Organizational resilience discourse before and after the pandemic. COVID-19, coronavirus disease.

Article quotes (examples)	
Before 2020	2020-2022
**Theme 1: Response to external threats**	
***Responses of businesses in disaster*** *“… .may affect their ability to adapt and thereby maintain resilience. In particular, adaptation to adversity stemming from the natural* *environment.”* ( Clément & Rivera, 2017)	***Resilience during the COVID-19 pandemic*** *“… resilient of the people and the sector they are working in becomes even more significant for the recovery of the business of this pandemic …”* ( Pathak & Joshi, 2021)
**Theme 2: Preparedness and organizational reliability**	
***Disaster mitigation planning*** *“… organizations can become wiser by looking at incidents outside their own sector and by using these recurring themes to explore the resilience of their emergency plans.”* ( Crichton *et al.*, 2009)	***Managerial policies and practices during Covid-19** “… system management and corporate social responsibility all played a positive role in this organisation's response to the outbreak of COVID-19.”* ( Yang *et al.*, 2021)
**Theme 3: Coping with occupational and job demands**	
***Human capital for organization resilience*** *“… business environment requires flexibility, innovation, and speedto-market, and companies must effectively develop and manage employees’ knowledge, experiences, skills, and expertise … for sustained organizational performance”* ( Manuti, 2014)	***Leadership role during a crisis*** *“… as a pillar to support organizational performance and survival throughout crises by nurturing an adaptive culture, through proper vision communication to all people and followers”* ( Madi Odeh *et al.*, 2021)
***Theme 4: Renewal and strategic agility and crisis as opportunity***	
***Strategy reformulation for business resilience*** *“Formalisation of strategic planning has been linked with long-term business performance and resilience formalisation activities are associated with improved responses to an acute interruption.”* ( Herbane, 2019)	***Beyond firm-centered sustainability*** *“SMEs can pursue transformative approaches to sustainability that are more environmentally, socially, and economically sustainable and better able to withstand shocks like the COVID-19 pandemic and can be significant contributors to community resilience.”* ( DiBella *et al.*, 2022)
**Theme 5: Supply chain vulnerabilities and disruptions**	
***Vulnerabilities towards natural disaster*** *“For example, Intel claimed a 1 billion loss in sales due to a reduction in computer manufacturing after floods in Thailand caused a shortage in hard drives needed to build the machines. In another situation, a fire in a semiconductor plant in New Mexico …” …* ( Stafford & Bouwens, 2016).	***Supply chain resilience in COVID-19 era*** *“… to swiftly recover from the COVID-19 pandemic's adverse impact … The findings of the study revealed that the lack of flexibility is the most critical causal barrier to building a resilient supply chain. Lack of planned resource management was also found to be an influential barrier.”* ( Banerjee *et al.*, 2022)
**Theme 6: Ensuring IT/IS/cyber security**	
***IS resilience in dealing with cyberattacks*** *“The rapid increase in the variety and complexity of cyber-attacks continues to challenge the ability of organizations to protect themselves from losses due to cyber intrusion.”* ( Bouwens & Stafford, 2019)	***COVID-19 pandemic or large-scale cyberattacks*** *“Globalisation and hyperconnectivity affect organisational resilience with threats such as the recent COVID-19 pandemic or large-scale cyberattacks.”* ( Marquez-Tejon *et al.*, 2022)
**Theme 7: Defining and conceptualizing resilience**	
***Resilience towards natural event*** *“This paper proposes a comprehensive conceptual framework of organizational adaptation and resilience to extreme weather events for addressing the effects of ecological discontinuities in organizational research and strategic decision-making.”* ( Linnenluecke *et al.*, 2012)	***Resilience towards disruption including pandemic*** *“… develop a holistic resilience framework and its contributing factors for organizations in the hospitality and tourism industry for coping with uncertain environments, such as those brought about by the COVID-19 pandemic.”* ( Ho *et al.*, 2022)

## Discussion

The study results show how organizational resilience was discussed before and after the pandemic in diverse industries. The themes discussed in the literature have also been shown in various publications, grouped into seven main themes, which are then identified as the discourse that occurs in each theme, as shown in Table 4. The discourse in each of these themes is then discussed in more detail in the following subsections.

### Response to external threats

The number of studies on organizational resilience in response to external threats has increased since the COVID-19 pandemic. Before 2020, studies on organizational resilience in this theme were limited to one main sub-theme: organizational response to nature-based exposure and disasters. For instance, some studies focused on natural disasters, including climate change and extreme weather ( Tisch & Galbreath, 2018), and floods ( Bang *et al.*, 2019). Nature-based exposure deals with climate change, which is considered a severe issue for companies, for it affects how a business entity will determine products and approach its customers. Mitigating natural disruptions can be aided by using a decision support system to help minimize the impacts ( Gumbira & Harsanto, 2019).

Moving forward in 2020 and beyond, numerous organizational resilience studies relate to the COVID-19 pandemic. This sub-theme is dominant and found in more than ten papers of our dataset. For instance, a study by Herrero and Kraemer (2022) examines how non-profit fundraisers dealt with a sudden decline in financial revenue during the pandemic by creating strategic ways of raising money. Furthermore, during the pandemic, Țiclău *et al.* (2021) interviewed ten female leaders from the non-profit and private sectors, revealing that government regulation, support, and financial pressures have been the major organizational challenges regardless of the sector. By contrast, the organizational dimension seems to impact the ability to adapt and respond to adversity. One study further discovered that organizational resilience and leadership skills play a crucial role in how well private higher education institutions succeed, even in times of crisis like the COVID-19 outbreak ( Zahari *et al.*, 2022). Based on our review of this theme, it can be implied that COVID-19 has even attracted more researchers to study organizational resilience, thus contributing to this literature using profit- and non-profit-oriented entities.

Preparedness and organizational reliability

This study’s results reveal that the theme of preparedness and organizational reliability contributes most to the dataset. Furthermore, the proportion of this theme before and after the pandemic is relatively balanced. The studies conducted before the pandemic deal with various topics related to the preparedness and organizational reliability theme, such as the significance of emergency planning for enhancing business resilience ( Crichton *et al.*, 2009), employee stock ownership and employee governance involvement for boosting resilience ( Lampel *et al.*, 2014), and building organizational resilience train through balancing organizational structures ( Andersson *et al.*, 2019). We also found studies with an emphasis on corporate social responsibility (CSR) embeddedness ( Lamprinakis, 2019) and strategic human resource management ( Al-Ayed, 2019), which contribute to organizational resilience.

In 2020 and beyond, studies on this theme have incorporated the COVID-19 pandemic as one of the research settings. For instance, in a study by Yang *et al.* (2021) employing the retail industry in China, it was uncovered that various factors, such as supply chain, digital construction, improvisational capability, and CSR had a positive role in responding to the COVID-19 outbreak. Meanwhile, Orth and Schuldis (2021) put forward organizational learning by implementing an open system culture to enable employees to withstand adversity, such as the COVID-19 pandemic. Furthermore, Rai *et al.* (2021) emphasize the essence of having the capability to predict the crisis and build robustness and recoverability since they positively affect the social and economic aspects of firm sustainability. In addition, an organization’s reliability during a crisis is also affected by its business network and social capital ( Chowdhury *et al.*, 2019; Ozanne *et al.*, 2022).

### Coping with occupational and job demands

The theme of ‘coping with occupational and job demands’ shows a balanced proportion between before and after 2020. Studies on this theme before 2020 are dominant on the general topics related to the role of conducive organizational culture ( Ahiauzu & Ololube, 2017) and sound psychological condition ( Cho *et al.*, 2017) in fostering firm resilience. Other topics related to the relationship of various factors with organizational resilience are also present, such as citizenship behavior ( Paul *et al.*, 2019), organizational learning ( Abu-Tineh, 2011), and human resource practices ( Bustinza *et al.*, 2019).

Like the previous themes, in 2020 onwards, the studies on ‘coping with occupational and job demands’ also mix with the COVID-19 context. For example, a study by Fathy El Dessouky and Al-Ghareeb (2020) proposes that an organization must support its workforce to be resilient in a dynamic environment such as an outbreak. Furthermore, using a case study of Geneve airport in Switzerland, a study by Romig (2021) emphasizes the importance of assessing the current contingency plans and devising fresh strategies to lessen the enormous operational and financial effects of the unheard-of circumstance, such as COVID-19 pandemic. In the context of the hotel industry, a study by Prasongthan (2022) revealed that organizational resilience positively affects employee engagement in the company, even during the outbreak, which implies that companies must explore various ways to enhance their employees’ resilience.

### Renewal and strategic agility and crisis as opportunity

This theme relates to various efforts made by organizations to recover themselves from unwanted circumstances, such as crises. Our dataset on this theme also shows a balanced proportion before and after the pandemic. Before 2020, the studies on this theme primarily discussed various efforts made by organizations to build resilience through the implementation of business reputation continuity ( Koronis & Ponis, 2012), information and technology optimization ( Chewning *et al.*, 2012; Mazini, 2014), dynamic capabilities ( Jiang *et al.*, 2019), the implementation of strategic human resource management ( Bouaziz & Smaoui Hachicha, 2018), relational leadership ( Teo *et al.*, 2017), and affect and team leadership ( Sommer *et al.*, 2016).

The theme in 2020 and beyond certainly captures the COVID-19 outbreak because it has altered how organizations view their current strategies to stay competitive. A dominant topic on this theme relates to the essence of organizational learning. For instance, a study by Zhou and Sun (2020) focuses on a red teaming strategy to encourage organizational learning and resilience. Using Huawei company as a case study, they argued that the red teaming strategy helps Huawei learn from its industry counterparts and ensures that the company operates robustly in the face of a challenging external environment. Furthermore, a study on the primary care sector conducted by Schuttner e *t al.* (2021) reveals that developing organizational learning and firm resilience may be a helpful step in implementing continuous quality improvement agenda within a company. Besides, a brief paper by independent authors reports that focusing on organizational learning at the individual, group, and collective levels can help a company become more resilient in the face of adversity and turn potentially destructive situations into beneficial sources of opportunity (“How Organizational Learning Increases Organizational Resilience: Build Multi-Stakeholder Networks to Strengthen the Effect,” 2020). Future discussions on renewal and strategic ability can connect resilience and sustainability or its specific aspects, such as sustainability innovation. Organizations can strategically build a strong foundation for long-term success, reduce risk exposure, and enhance their ability to adapt to change ( Harsanto *et al.*, 2018; Harsanto & Permana, 2021).

### Supply chain vulnerabilities and disruptions

This theme experienced an increase before and after the COVID-19 pandemic compared to other themes. The supply chain is one of the areas that has been severely disrupted and has produced many publications that examine the impact of the pandemic on the supply chain, predictions of future disruptions, and various steps to build supply chain resilience for organizations ( Nikolopoulos *et al.*, 2021). The discourse that occurred on this theme before and after the pandemic was the focus of general pre-pandemic investigations on natural disasters with topics related to safety performance indicators ( Rigaud & Martin, 2014), the influence of culture and resilience on supplier risk levels ( Borekci *et al.*, 2014; Mandal, 2017), resilience to the buyer-supplier relationship ( Yilmaz Borekci *et al.*, 2015), vulnerabilities as a consequence of the currently developing global supply chain ( Stafford & Bouwens, 2016), as well as the influence of supply chain agility and resilience towards performance ( Altay *et al.*, 2018). Regarding supply chain vulnerabilities due to global operations, Stafford and Bouwens (2016), for example, investigate how the flood in the case of Intel in Thailand or the fire in New Mexico significantly impacted the company’s supply chain globally.

While after the pandemic, the focus of this theme is on vulnerabilities and disruptions due to the COVID-19 pandemic in diverse industrial contexts, such as healthcare ( Hundal *et al.*, 2021) and Small and Medium Enterprises (SMEs) ( Banerjee *et al.*, 2022). The challenges of the pandemic have encouraged the birth of publications that contribute to building a culture of resilience within organizations, such as stakeholder partnerships, systematic sharing, and rapid responses, including the Strategic Management of Organizational Resilience (SMOR). Classic techniques such as Lean Six Sigma can also be used to build resilience ( Hundal *et al.*, 2021). Apart from that, organizational ambidexterity, which is concerned with balancing exploitation and exploration along with solid collaboration, is also an effort to build supply chain resilience and agility in the long term ( Küffner *et al.*, 2022).

### Ensuring IT/IS/cyber security

This theme was discussed in the literature before and after the pandemic with relatively the same intensity and tended to decrease slightly (-0.95%). Before the pandemic, the discussion in the publications was about building resilience, especially in the area of information systems in dealing with the risk of cyberattacks to the organizations that invest in hardware and software in their information systems, by identifying various potential sources for IS vulnerabilities at various levels, starting from individual to organizational. Various tools and strategies were developed to increase organizational resilience in various cyber-attack phases, including recovery, access gain, escalating privileges, system browsing, and installing additional tools ( Bouwens & Stafford, 2019). This topic is also related to industry 4.0, marked by the cyber-physical environment ( Nica, 2019).

After the pandemic, this discourse theme is still about resilience in dealing with cyberattacks, with the possibility of bigger cyberattacks equivalent to the challenges of the COVID-19 pandemic. The direction of the discussion is regarding globalization characterized by super-high connectivity, making IT and company operations efficient and vulnerable simultaneously. Here, enterprise security risk management (ESRM) as a security management approach emphasizing collaboration between business and security professionals becomes essential in mitigating existing risks ( Marquez-Tejon *et al.*, 2022). In organizations, the need for resilience is also considered by standardization institutions such as ISO, which is reflected in ISO 22316, defining organizational resilience with the keywords absorb and custom in changing environments. Resilient organizations can anticipate and respond appropriately, including allocating the right resources to various internal and external threats ( Bennett & Lemoine, 2014).

### Defining and conceptualizing resilience

This theme did not experience an increase during the pandemic compared to before the pandemic. Of course, in terms of the period, it is not comparable between before the pandemic (before 2020) and after the pandemic (2020 onwards). However, several other themes in a short time (2021-2022) reveal that publications can exceed what happened in the past or before the pandemic. In line with other themes, the discourse on this theme before the pandemic was more directed toward efforts to build resilience in dealing with disruptions from the natural environment. This conceptualization is carried out from various perspectives, for example, from the organization’s capacity, which includes cognitive behavioral and contextual ( Lengnick-Hall *et al.*, 2011), business model innovation ( Müller *et al.*, 2018), and layers of micro, meso, macro ( Luo & Shi, 2011) in various natural disasters such as floods, hurricanes or heat waves ( Linnenluecke *et al.*, 2012). This conceptualization is necessary because the needs for resilience in each organization are different. Resilience is highly imperative for high-reliability organizations (HROs) such as hospital or fire department units ( Ishak & Williams, 2018). The proposed concept, for example, is the Resilience Architecture Framework (RAF), which consists of ambidexterity, dynamic capabilities, rigidity ( Limnios *et al.*, 2014), or three dimensions of resilience, including anticipation, coping, and learning ( Duchek, 2014), or the three aspects or organization resilience including power structure, actions, language ( Witmer, 2019). Resilience conceptualization is also carried out with other related concepts such as adaptation, change, networks, systems, social capital, or vulnerability ( Hall *et al.*, 2017).

After the pandemic, the definition and conceptualization of resilience continued with a discourse emphasis on the need for severe disruption due to COVID-19. The proposed framework, for example, includes the mindset and action elements needed to build resilience in one of the sectors worst affected by the pandemic: tourism and hospitality ( Ho *et al.*, 2022). This sector is also sensitive during the pandemic to disruptions originating from natural disasters ( Chowdhury *et al.*, 2019; Prayag *et al.*, 2020). With the right mindset and action, it is expected that the organization will be able to bounce back and undergo a fast recovery. Indeed, this is a difficult challenge because of the different shock intensities in volume, velocity, or variety ( Cruickshank, 2020). Resilience is considered a meta-capability consisting of various individual constructs that go through various stages of crisis ( Duchek, 2020). Among the interesting conceptualizations is the effort to measure resilience using quantitative measures involving time windows and counter-factuality ( Ilseven & Puranam, 2021).

**Figure 2.**
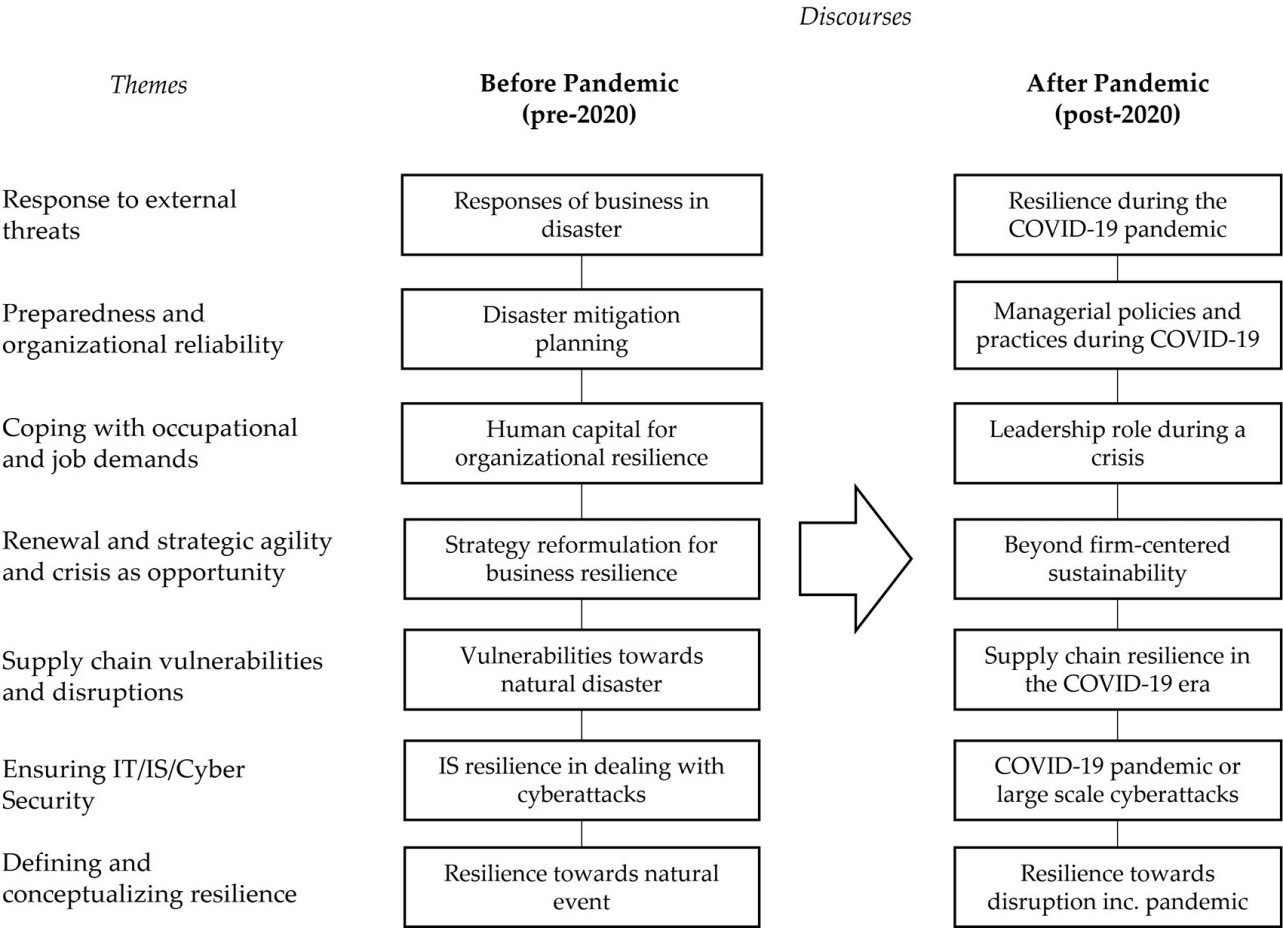
Summary of discourses (themes adopted from Denyer, 2017). COVID-19, coronavirus disease.

Figure 2 illustrates the discourse summary before and after the pandemic. Among the seven analyzed themes, each has distinct discourse but with a common theme of pre-pandemic focus on resilience to natural disasters, while post-pandemic discourse has shifted to the challenges posed by the COVID-19 pandemic.

## Implications and future directions

This study is of interest to both academics and practitioners. Academically, this study advances knowledge of organizational resilience by providing a discourse analysis before and after the pandemic. Before the pandemic, researchers focused primarily on natural disasters such as floods or fires, with pandemics receiving little attention. However, the pandemic has since garnered significant attention, substantially impacting the global socio-economic landscape. Practitioners and policymakers can direct efforts to build organizational resilience in the face of diverse types of disruptions, including huge ones like pandemics.

This study has several limitations that open up avenues for future research. First, the study finds that the main focus of attention in this area is on a few sectors and suggests that future research should pay more attention to other sectors, such as retail, construction, or textile, which have not yet received much attention. Second, technically, the academic document search in this study was limited to the Scopus database, which may result in some academic documents being overlooked. Future studies could complement the Scopus database with other academic databases. Third, since this current study only employs a search strategy on the title field, future studies may use a broader search strategy by adding abstract and keyword fields.

Further, future discourse studies can build upon the themes discussed in this study by exploring each of these themes in greater depth. For example, future research can focus on the evolution of resilience conceptualization before and after the pandemic and beyond. Besides, quantitative studies employing statistical analysis may also be conducted in future studies to gain insight into organizational resilience.

## Data Availability

Figshare: 273 document results Organizational Resilience.csv., https://doi.org/10.6084/m9.figshare.23567103 ( Harsanto & Firmansyah, 2023b). This project contains the following underlying data:
- 273 document results Organizational Resilience.csv (The dataset contains 273 list of documents indexed in Scopus mentioning “organizational resilience” in either title, keyword, or abstract. The dataset is specific on business and economics fields only.) Data are available under the terms of the Creative Commons Attribution 4.0 International license (CC-BY 4.0).
