# Supplementary material for: The discourse of organizational resilience before and after the global pandemic
Source: F1000Res. 2023 Sep 11;12:1128. [Version 1] doi: 10.12688/f1000research.133601.1 (PMC10955188; doi:10.12688/f1000research.133601.1)
Supplement: Peer review history

# Reviewer report: Reviewer response for version 1

**DOI:** 10.5256/f1000research.146607.r247507

**Published:** 2024-03-20

**Authors:** Diego Polanco-Lahoz

**Affiliations:** 1 Texas Tech University, Lubbock, Texas, USA

Summarized, the paper analyzes using thematic analysis the change in trends and how was understood Organizational Resilience in the media and other sources, before and after the COVID-19 pandemic. The thematic analysis shows that most vulnerabilities are related to the supply chain and that preparedness is an important topic to consider. This helps the literature by giving value and a time-lapse to the changes in understanding of Organizational Resilience.

 I think the paper is good. It does not offer a breakthrough of new ideas or concepts, but papers such as those analyzing the change of conceptualization and how people understand topics are also important. The methodology used is precisely good. Thematic analyses are needed when the data to be analyzed is qualitative, and in this case, this was the vast majority. The only enhancement I could see is the motivation behind it. When studies talk about COVID-19 is clear where the motivation comes through, but also many of those types of drafts do not state explicitly why the proposal study is important. As a researcher in the field, I get it, but not all readers are researchers with years of reading in the field. Besides that, detail, and some formatting issues, the paper is good for approval. I hope the authors are proud of the work done!

 Shown in detail, the introduction shows a great starting point for an Organizational Resilience (OR) standpoint just before COVID-19. The awareness grew even higher when the pandemic was at its peak. The major point to consider in this section is the origin of the concept. Future papers trying to go beyond the literature and explore the origin of OR should be aware of initial management theories as well as the ecological perspective of resilience. The methodology section is good, and I have used similar phrases when looking for the data. The only detail that readers should be aware of is the software tool to analyze data. When researchers find thousands of words and results, a better approach could be using NVivo with its internal tool of thematic analysis. The results are shown perfectly in detail, I cannot include anything in this section that has not been shown. The tables presented help to understand the idea of each paragraph. In the discussion, all sections are very well presented, the only major point is related to the definition of OR. It seems logical that the discussion needs to present information derived from the methodology results, but the topic of OR conceptualization goes beyond 2022 which is the last study specified. If we consider the literature there are multiple ways to conceptualize OR: as a process (Duchek, 2020), as a static capability (Chen et al., 2021), or as an outcome (Ilseven and Puranam, 2021). These three perspectives are currently my primary focus of research related to OR. Future literature will converge when the phenomena of OR understanding can be classified more clearly, and that will be the time to consider which approach is better depending on the context that is being seen. Things such as the stated before are needed for the reader to consider if OR is part of their research goal.

 Again, going over the details of definitions and obvious phenomena derived from understanding OR in all its extensions, the paper is very good and helps literature with the understanding before and after COVID-19. Very good job!

Is the work clearly and accurately presented and does it cite the current literature?

Yes

If applicable, is the statistical analysis and its interpretation appropriate?

Yes

Are all the source data underlying the results available to ensure full reproducibility?

Yes

Is the study design appropriate and is the work technically sound?

Yes

Are the conclusions drawn adequately supported by the results?

Yes

Are sufficient details of methods and analysis provided to allow replication by others?

Yes

Reviewer Expertise:

Organizational Resilience in Higher Education

I confirm that I have read this submission and believe that I have an appropriate level of expertise to confirm that it is of an acceptable scientific standard.

# Reviewer report: Reviewer response for version 1

**DOI:** 10.5256/f1000research.146607.r227892

**Published:** 2023-12-15

**Authors:** Naglaa Fathy El Dessouky

**Affiliations:** 1 University of Bahrain, Zallak, Bahrain

- In general, the article tackled an important up to date topic.
- The paper succeeded in presenting a thorough literature review about the topic.
- The paper employed an adequate research methodology. However, it was not very reliable that the authors search for documents for the study’s dataset in only one day stated on July 5, 2022. Moreover, the authors’ search in Scopus focused only on Business and Economics without providing detailed arguments about the reason of selection while other dataset can be an asset for the study such as Management and Public Policy.
- The study succeeded in presenting the results and discussion in a systematic way.
- The author may improve the implications of the study by providing a concrete action plan for the public policy makers and the management of organizations.

Is the work clearly and accurately presented and does it cite the current literature?

Yes

If applicable, is the statistical analysis and its interpretation appropriate?

Yes

Are all the source data underlying the results available to ensure full reproducibility?

Yes

Is the study design appropriate and is the work technically sound?

Yes

Are the conclusions drawn adequately supported by the results?

Yes

Are sufficient details of methods and analysis provided to allow replication by others?

Yes

Reviewer Expertise:

Public Policy Analysis and Evaluation, Management Sciences, Organizational studies, Sustainability, Virtual Organizations.

I confirm that I have read this submission and believe that I have an appropriate level of expertise to confirm that it is of an acceptable scientific standard.
